# The effect of selenium supplementation on sonographic findings of salivary glands in papillary thyroid cancer (PTC) patients treated with radioactive iodine: study protocol for a double-blind, randomized, placebo-controlled clinical trial

**DOI:** 10.1186/s13063-023-07470-2

**Published:** 2023-08-07

**Authors:** Sepide Amini, Marjan Golshani, Masoud Moslehi, Somayeh Hajiahmadi, Gholamreza Askari, Bijan Iraj, Mohammad Bagherniya

**Affiliations:** 1 Nutrition and Food Security Research Center and Department of Community Nutrition, School of Nutrition and Food Science, Isfahan University of Medical Sciences, Isfahan, Iran; 2 Department of Internal Medicine, School of Medicine, Isfahan University of Medical Sciences, Isfahan, Iran; 3 Department of Medical Physics, School of Medicine, Isfahan University of Medical Sciences, Isfahan, Iran; 4 Department of Radiology, Isfahan University of Medical Sciences, Isfahan, Iran; 5 Anesthesia and Critical Care Research Center, Isfahan University of Medical Sciences, Isfahan, Iran; 6 Isfahan Endocrine and Metabolism Research Center, Isfahan University of Medical Sciences, Isfahan, Iran

**Keywords:** Selenium, Thyroid cancer, Radioactive iodine, Salivary glands, Supplementation

## Abstract

**Background:**

Thyroid cancer is a very damaging disease. The most common treatment for this disease includes thyroidectomy and then using radioactive iodine (RAI). RAI has many side effects, including a decrease in salivary secretions, followed by dry mouth and oral and dental injuries, as well as increased inflammation and oxidative stress. Selenium can be effective in these patients by improving inflammation and oxidative stress and by modulating salivary secretions. So far, only one clinical trial has investigated the effect of selenium on thyroid cancer patients treated with radioiodine therapy (RIT) conducted on 16 patients; considering the importance of this issue, to show the potential efficacy of selenium in these patients, more high-quality trials with a larger sample size are warranted.

**Methods:**

This is a parallel double-blind randomized controlled clinical trial that includes 60 patients aged 20 to 65 years with papillary thyroid cancer (PTC) treated with RAI and will be conducted in Seyyed al-Shohada Center, an academic center for referral of patients to receive iodine, Isfahan, Iran. Thirty patients will receive 200 µg of selenium for 10 days (3 days before to 6 days after RAI treatment) and another 30 patients will receive a placebo for the same period. Sonographic findings of major salivary glands, salivary secretions, and sense of taste will be evaluated before and 6 months after 10-day supplementation.

**Discussion:**

Due to its anti-inflammatory and antioxidant effects, as well as improving salivary secretions, selenium may improve the symptoms of thyroid cancer treated with radioactive iodine. In past studies, selenium consumption has not reduced the therapeutic effects of radiation therapy, and at a dose of 300 to 500 μg/day, it has not had any significant side effects in many types of cancer under radiation therapy.

**Trial registration:**

Iranian Registry of Clinical Trials IRCT20201129049534N6. Registered on 16 September 2021.

**Supplementary Information:**

The online version contains supplementary material available at 10.1186/s13063-023-07470-2.

## Administrative information

Note: the numbers in curly brackets in this protocol refer to the SPIRIT checklist item numbers. The order of the items has been modified to group similar items (see http://www.equator-network.org/reporting-guidelines/spirit-2013-statement-defining-standard-protocol-items-for-clinical-trials/).

**Table:** 

Title {1}	The effect of selenium supplementation on sonographic findings of salivary glands in papillary thyroid cancer (PTC) patients treated with radioactive iodine: study protocol for a double-blind, randomized, placebo-controlled clinical trial.
Trial registration {2a and 2b}	IRCT20201129049534N6; 16 September 2021.
Protocol version {3}	Version 2, December 2022
Funding {4}	Isfahan University of medical sciences; Grant number: 240096
Author details {5a}	Sepide Amini^1^, Marjan Golshani^2^, Masoud Moslehi^3^, Somayeh Hajiahmadi^4^, Gholamreza Askari^1,5^, Bijan Iraj^6^, Mohammad Bagherniya^1,5^ ^1^Nutrition and Food Security Research Center and Department of Community Nutrition, School of Nutrition and Food Science, Isfahan University of Medical Sciences, Isfahan, Iran ^2^Department of Internal Medicine, School of Medicine, Isfahan University of Medical Sciences, Isfahan, Iran. ^3^Department of Medical Physics, School of Medicine, Isfahan University of Medical Sciences, Isfahan, Iran ^4^Department of Radiology, Isfahan University of Medical Sciences, Isfahan, Iran. ^5^Anesthesia and Critical Care Research Center, Isfahan University of Medical Sciences, Isfahan, Iran. ^6^ Isfahan Endocrine and Metabolism Research Center, Isfahan University of Medical Sciences, Isfahan, Iran.
Name and contact information for the trial sponsor {5b}	Isfahan University of medical sciences Postal Code: 81746–73461 Tell: (+ 98)-31–3668-0048
Role of sponsor {5c}	Financial support and supervision

## Introduction

### Background and rationale {6a}

Thyroid cancer (TC) is more common than other endocrine malignancies and its incidence is increasing worldwide [1, 2]. TC includes three main tumor types with different histologic and clinical characteristics: differentiated thyroid carcinoma (DTC), anaplastic (undifferentiated or poorly differentiated) thyroid carcinoma (ATC), and medullary thyroid carcinoma (MTC). DTC is the most common type of thyroid cancer and has a better prognosis than others [3, 4].

One of the most important causes of thyroid cancer is environmental factors such as ionizing radiation, toxic chemical compounds, and halogenated pesticides. The amount of consumed iodine is also effective on the probability of contracting it, so both deficiency and excess consumption of iodine can lead to an increase in the probability of contracting thyroid cancer [5, 6]. Some modifiable individual factors, including weight, are also related to the possibility of contracting this type of cancer [7]. Inflammation is also an effective factor in causing and aggravating thyroid cancer. Generally, in thyroid cancer, there is an increase in inflammatory factors such as Nuclear factor kappa light chain enhancer of activated B lymphocytes (NF-κB) and C-Reactive Protein (CRP), which in turn causes the tumor to progress and worsen the patient’s condition [8–10].

Current treatments for thyroid cancer include thyroidectomy (complete or partial removal of the thyroid gland), tyrosine kinase inhibitors (TKI), and radioactive iodine (RAI). Among these, one of the most common methods includes thyroidectomy followed by the use of radioactive iodine [11–13]. The reason for using radioactive iodine is to drain thyroid remnants and control metastatic conditions [11, 13]. But it also has many side effects. One of the most important complications caused by radioiodine therapy (RIT) is its excessive entry into the salivary glands, which causes swelling and dysfunction of these glands, and as a result, saliva secretion decreases [14–19]. Following the decrease in saliva, dry mouth, and taste problems occur, which greatly affect the patient’s quality of life. Also, the possibility of tooth decay and gingivitis (gum inflammation) increases. If there is a long-term and chronic decrease in saliva, fibrosis of the oral mucous cells may even occur [20–24]. Another side effect of RIT is the creation of oxidative stress, which favors cancer recurrence [25]. It can also cause a disturbance in the modulation of inflammatory factors [26]. According to European guidelines, it is recommended to consume sour candy or chewing gum after RIT for hydration and to reduce the problems caused by decreased salivation [27], but many patients suffer from dry mouth and related problems even despite this practice [28].

Selenium is one of the essential minerals, the largest amount of which per gram of tissue is present in the thyroid gland and it affects thyroid health through its role in the structure of selenoproteins [29–31]. Selenoproteins play a role in the metabolism of thyroid hormones and the antioxidant system [32]. Selenium can help inhibit thyroid neoplasm, which is a potential effect through induction of apoptosis, stimulation of tumor suppressor genes, and antioxidant and anti-inflammatory function [33–37].

Recently, a clinical trial study investigated the effect of selenium on thyroid cancer patients undergoing RIT therapy, which reported that 300 μg/day of selenium from 3 days before to 6 days after RIT can help maintain salivary gland function [38]. This is the only trial study regarding the effect of selenium supplementation on patients with thyroid cancer under RIT treatment, which has a limited sample size (*n* = 16) [38], so this study will be conducted with a larger sample size to examine this issue.

### Objectives {7}

We aim to investigate the effects of selenium supplementation on sonographic findings of salivary glands in papillary thyroid cancer (PTC) patients treated with radioactive iodine. If this trial confirms our hypothesis, selenium supplementation may be used to improve the effects of existing treatments.

### Trial design {8}

A randomized, parallel, two-arm, double-blind, and placebo-controlled superiority clinical trial will be implemented.

## Methods: participants, interventions, and outcomes

### Study setting {9}

Our study will be performed at Seyyed al-Shohada Center, an academic center for the referral of patients to receive iodine, affiliated with Isfahan University of Medical Sciences, Isfahan, Iran.

### Eligibility criteria {10}

#### Inclusion criteria

- Age between 20 and 65 years
- Diagnosis of PTC with pathological findings of thyroid tissue biopsy.
- Treated with radioactive iodine

#### Exclusion criteria

- Suffering from sialadenitis (inflammation of salivary glands)
- Suffering from Sjogren’s syndrome
- Subjects with severe side effects after thyroidectomy who were not possible for RIT
- Collagen vascular disease involving the salivary glands.
- Any surgery on the salivary glands
- Pregnancy and breastfeeding
- Lack of patient permission
- Consumption of food supplements in the last 3 months (Consumption of any herbal supplement, for example, curcumin, ginseng, etc., or/and any vitamin or mineral supplement, or/and any other food supplement, including omega-3, etc.).
- Suffering from special diseases such as diseases caused by congenital diseases, immune system defects, etc.
- Report any adverse effects after taking supplements
- If there is a significant change in any of the patient’s treatments during the period of intervention by the relevant doctor

#### Reason for eliminating the data

- Consumption of less than 80% of selenium supplements by the patient.

### Informed consent procedures {26a}

In this study, after providing written and oral explanations about the objectives and method of conducting the research, written informed consent will be obtained from the patients if they wish to cooperate in the study.

### Additional consent provisions for collection and use of participant data and biological specimens {26b}

The consent form received from the patients includes their agreement to use the data in related future studies. Of course, patients can only agree to the current study and not accept future studies.

## Interventions

### Explanation for the choice of comparators {6b}

While receiving standard treatment, patients will be placed in one of two groups receiving a selenium supplement or placebo. The treatment group will receive one selenium capsule containing 200 µg of selenium per day and the control group will receive one placebo capsule containing 200 µg of maltodextrin per day for 10 days (3 days before to 6 days after radioactive iodine treatment).

The reason for choosing 200 µg is that a dosage of 200 µg/day per extra-dietary supplementation of selenium is generally considered safe and adequate for an adult of average weight [39]. In addition, the supplements available in the Iranian market have a dose of 200 μg per tablet. Also, Lee et al.’s study [38], which is somewhat similar to our study, used a dose of 300 μg. Therefore, 200 μg is close to this dose.

### Intervention description {11a}

Both treatment and placebo groups will be treated with radioactive iodine. The treatment group will receive one selenium capsule containing 200 µg of selenium per day for 10 days (3 days before to 6 days after radioactive iodine treatment). The control group will receive a placebo capsule containing 200 µg of maltodextrin per day for 10 days (3 days before to 6 days after radioactive iodine treatment). Selenium capsules are purchased from 21st Century company and the placebo is prepared by the Faculty of Pharmacy of Isfahan University of Medical Sciences. During the intervention, the type and dosage of the drugs used by the patients will not be changed, and the patients will receive all their usual treatments according to the previous routine (under the supervision of a specialist endocrinologist), and receiving selenium or placebo is merely a supplement to the common treatments they receive.

### Criteria for discontinuing or modifying allocated interventions {11b}

Each participant will be allowed to withdraw from the study at any time. Also, the reasons for each individual’s withdrawal from the study will be explained in detail in the study results.

### Strategies to improve adherence to interventions {11c}

Selenium and placebo tablets will be delivered to the patients by the researcher (M.G) and during ten days of supplementation, the patients will be reminded to take the tablets by phone call. Also, patients will return the empty can of the supplement after ten days.

### Relevant concomitant care permitted or prohibited during the trial {11d}

The patient will receive all the usual care and treatment under the supervision of a specialist doctor. There will be no change in receiving standard care during the study. Patients only received selenium supplements or a placebo in addition to receiving routine treatments.

### Provisions for post-trial care {30}

All standard treatments will be continued after the study, but selenium or placebo supplements will only be received for 10 days during the study.

### Outcomes {12}

In general, the variables evaluated in this study will include demographic variables, assessment of disease severity, and evaluation of changes in sonographic findings of major salivary glands (including echogenicity, size, margin, and contour in sonography).

Demographic variables (including age, gender, marital status, smoking, medical history and medical history, education level, occupation, and use of supplements and drugs) will be collected from all participants by completing a general information questionnaire.

Before and 6 months after the 10-day intervention with selenium or placebo, sonography was taken from parotid and submandibular salivary glands and according to sonography, these salivary glands will be evaluated in terms of echogenicity, size, margin, and contour.

Huang et al.’s study including 446 patients with thyroid carcinoma, reported that the side effect of radioactive iodine on salivary glands occurs after a few months [40]. Also, Lee et al.’s study, which investigated the effect of selenium intake on reducing the side effects of radioactive iodine on salivary glands, examined the findings after 6 months [38]. Therefore, we will examine the changes in salivary gland sonographic findings after 6 months.

The level of taste sensation and secretion of salivary glands will be evaluated by the evaluation questionnaire of the level of sialadenitis [41]. This questionnaire contains 14 questions and each question can have between 1 and 4 scores (score 1, not at all; score 2, a few times; score 3, fairly often; and score 4, almost always.). Therefore, the overall score varies between 14 and 56 [41].

Before and 6 months after the 10-day intervention with selenium or placebo, 10 ml of blood will be taken from the patients and collected in plastic tubes. A centrifuge will be used to separate the serum samples. Then the serum will be poured into 0.5 ml microtubes. The samples will be stored in a freezer at − 70 °C. To reduce the error in the measurements, all blood samples will be taken at 8–10 am in the fasting state.

CRP will be evaluated at the beginning and end of the study, and if there will be no budget restrictions, we will also evaluate total antioxidant capacity (TAC) and superoxide dismutase (SOD).

### Participant timeline {13}

**Fig. 1:**
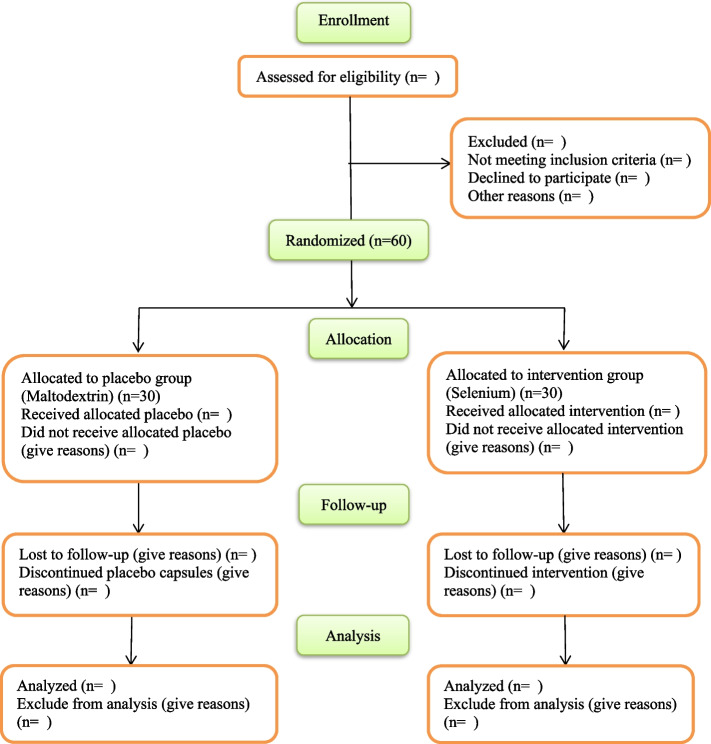
Participant flow diagram according to the Consolidated Standards of Reporting Trials (CONSORT) 2010 statement

**Fig. 2:**
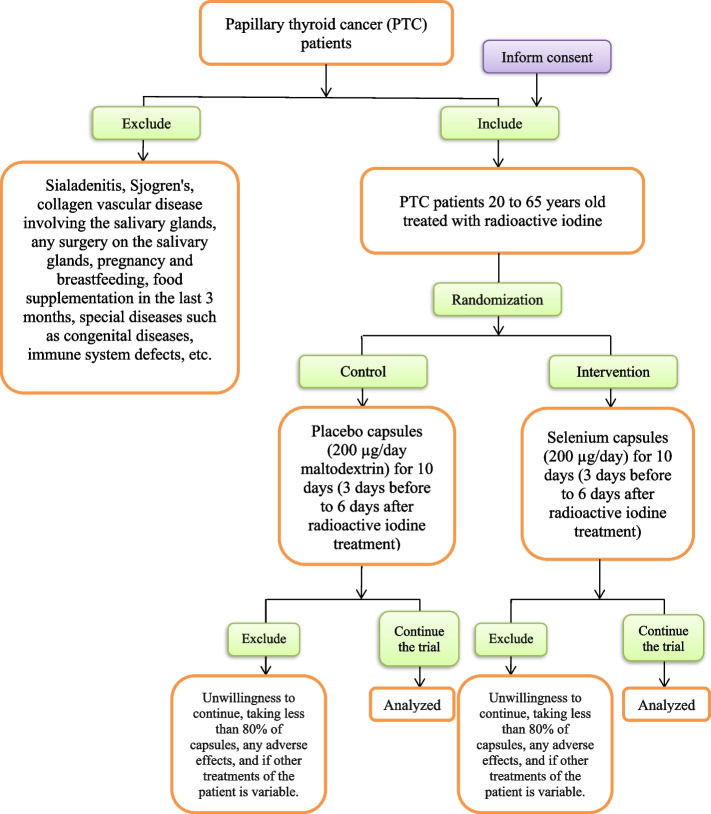
Trial procedure flow sheet

The study flow diagram is according to Table 1 and Figs. 1 and 2.

**Table 1:**
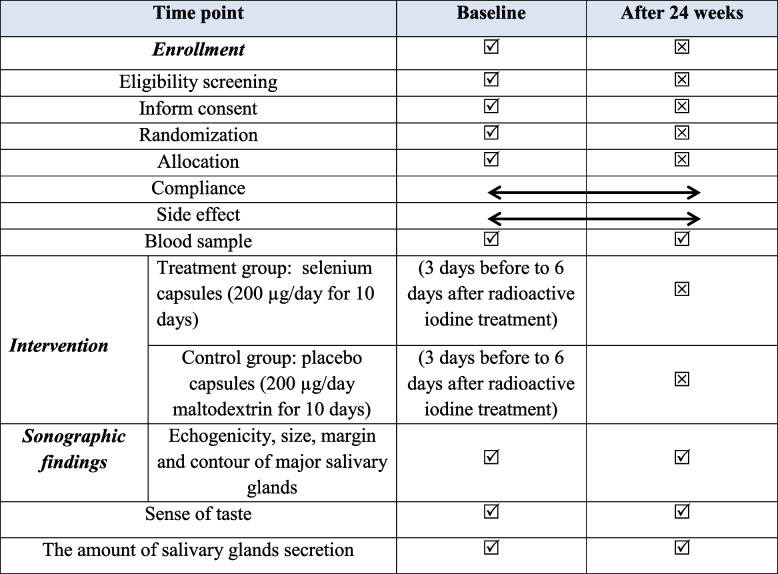
Timeline and applied tests

### Sample size {14}

Since our study is the first in its kind, we calculate the sample size base on CRP as one the most important parameters of this study. Based on a previous study using selenium supplementation on patients with inflammation to reduce inflammatory factors such as CRP [42], and considering 80% power, and the first type error α = 0.05 and the second type error β = 0.20, based on following equation sample size were calculated:

$$n\hspace{0.17em}=\hspace{0.17em}2[{(\mathrm{Z}1-\mathrm{\alpha }/2\hspace{0.17em}+\hspace{0.17em}\mathrm{Z}1-\upbeta )}^{2}\hspace{0.17em}\times \hspace{0.17em}{\mathrm{S}}^{2}]/{\Delta }^{2}\hspace{0.17em}\to \hspace{0.17em}2[{(1.96\hspace{0.17em}+\hspace{0.17em}0.84)}^{2}\hspace{0.17em}\times \hspace{0.17em}{(10.9)}^{2}]/{(8)}^{2}$$

A total of 60 subjects (i.e., 30 participants in each group) would be required.

### Recruitment {15}

Patients will be selected according to the entry and exit criteria from the patients who refer to Seyyed al-Shohada Center, an academic center for referral of patients to receive iodine, Isfahan, Iran.

## Assignment of interventions: allocation

### Sequence generation {16a}

After meeting the entry criteria, each patient will be randomly assigned to one of the two supplemental groups with selenium or placebo using the reliable random number generator website “https://www.sealedenvelope.com/simple-randomiser/v1/lists” and using a block size of four based on age and sex using the website https://www.sealedenvelope.com/simple-randomiser/v1/lists, with the ratio of 1:1. Sample patients will be 20 to 65 years old. Therefore, the age threshold of 20 to 65 years will be considered. For the randomization, the stratification factors are age (20 to 45, or 46 to 65) and sex (male or female).

### Concealment mechanism {16b}

Completely identical packages of selenium and placebo tablets with blinded labels A or B will be provided to the researcher without specifying the type of supplement. Researchers and patients will not know the type of supplement on each label until the end of the statistical analysis.

### Implementation {16c}

An independent reviewer will assign the participants to the intervention or control group, and also the generation of the allocation sequence of the patients will be monitored by the second researcher (B.I).

## Assignment of interventions: blinding

### People who will be blind {17a}

This will be a double-blind study, so the researchers and the subjects will not know which group they will enter. Also, selenium and placebo supplements will be produced in the same way in terms of shape, color, size, and smell, and they will have the same packaging.

### Procedure for unblinding if needed {17b}

The disclosure of the label of the supplements will only take place at the end of the study after the completion of the statistical analysis. In case of any unblinding during the study, it will be reported in detail in the Iranian Registry of Clinical Trials and the final results of the study.

## Data collection and management

### Plans for assessment and collection of outcomes {18a}

Major salivary glands (echogenicity, size, margin, and contour) will be examined by sonography before and 6 months after the 10-day intervention with selenium or placebo.

A questionnaire evaluating the degree of dry mouth and its effects on the daily life of patients will be used to evaluate the sense of taste and secretion of salivary glands.

### Plans to promote participant retention and complete follow-up {18b}

To encourage patients to cooperate as much as possible, it will be explained about the possible effects of selenium supplementation on the reduction of complications caused by the treatment of thyroid cancer patients with radioactive iodine.

### Data management {19}

The data will be carefully recorded and protected by a blind researcher. After completing the data, the result set will be reviewed.

### Confidentiality {27}

The ethical principles of confidentiality of the names and identification details of the patients will be fully maintained. The researchers will only mention the results of the study without mentioning the names and identification details of the patients, so the patients will not be identifiable under any circumstances.

### Plans for collection, laboratory evaluation, and storage of biological specimens for genetic or molecular analysis in this trial/future use {33}

After separating the serum from the collected blood samples, they will be immediately placed in a freezer at − 70°C until the tests will be performed. If the patient agrees, the blood sample will be stored for future studies. Genetic tests are not related to the present study.

## Statistical methods

### Statistical methods for primary and secondary outcomes {20a}

In this study, the number of quantitative variables will be reported as mean (standard deviation), and qualitative variables will be reported as number (percentage). The evaluation of the normality of the distribution of quantitative variables will be done using the skewness index and the Q-Q plot diagram. Intra-group analyses will be performed using paired *t*-test and between-group analyses will be performed using independent *t*-test and ANCOVA. The distribution of qualitative variables will be compared between two groups using the chi-square test. SPSS version 22 software will be used to analyze the data with a significance level of *P* < 0.05.

The primary results of this study include the evaluation of CRP and changes in sonographic findings of major salivary glands (including echogenicity, size, margin, and contour in sonography), and the secondary results include the evaluation of the level of taste sensation and secretion of salivary glands by the questionnaire to assess the level of sialadenitis [41].

### Interim analyses {21b}

Not applicable.

### Methods for additional analyses (e.g., subgroup analyses) {20b}

No subgroup analyses are planned.

### Methods in analysis to handle protocol non-adherence and any statistical methods to handle missing data {20c}

The analysis will be done in an intention-to-treat format so that the data from excluded patients are also mentioned in the results. The reason for each patient’s withdrawal, especially the presence of any side effects, will be mentioned in detail. A sensitivity analysis will also be done. Missing data will be checked by multiple imputations.

### Plans to give access to the full protocol, participant-level data, and statistical code {31c}

If there will be logical requests for data in the direction of the present protocol, the corresponding author will send the data set. Otherwise, all information will remain confidential.

## Oversight and monitoring

### Composition of the coordinating center and trial steering committee {5d}

The performance of the researchers and all stages of the study will be under the supervision of the Ethics Committee and the Vice-Chancellor of Isfahan University of Medical Sciences, so that if there is any contradiction with the ethical principles, the preservation of the patient’s health and also the preservation of his human values, the corrections or the termination of this study will be applied.

### Composition of the data monitoring committee, its role and reporting structure {21a}

Data monitoring will be done by the academic committee of Isfahan University of Medical Sciences, which includes impartial and continuous monitoring.

### Adverse event reporting and harms {22}

Any side effects, whether significant or minor, will be reported in full detail though previous studies have reported no adverse effects of selenium supplementation in several types of cancer treated with radiation therapy [43, 44].

### Frequency and plans for auditing trial conduct {23}

The present study will be supervised by the Ethics Committee of Isfahan University of Medical Sciences. Unexpected inspection of the accuracy and correctness of the study and the correct performance of the researchers will be done at least twice during the study.

### Plans for communicating important protocol amendments to relevant parties (e.g., trial participants, ethical committees) {25}

If there is a need for any changes in the study protocol, the changes will be made under the supervision and with the approval of the Ethical Committee of Isfahan University of Medical Sciences. All changes will be reported in detail at https://irct.ir/.

### Dissemination plans {31a}

Final data and results will be presented in official publications.

## Discussion

Thyroid cancer is a common disease worldwide [1, 2]. Inflammation is one of the most important factors causing and worsening thyroid cancer [8–10]. Also, oxidative stress factors are generally high in these patients and are effective in the relapse process [45–48]. The most common treatment solution for these patients is thyroidectomy and then treatment with radioactive iodine to drain thyroid remnants and control metastatic conditions [11–13]. But this solution has many side effects. For example, radioactive iodine, in addition to its beneficial effect on improving cancer, by excessively entering the salivary glands, reduces the secretion of saliva and the annoying mouth dryness [18, 20, 21, 23, 24]. The current solution to improve the salivary secretion of these patients is to use sour candy or chewing gum. But in many patients, this solution does not bring much improvement [27, 28]. Other side effects of RIT include increased oxidative stress and inflammation [26, 45].

**Fig. 3:**
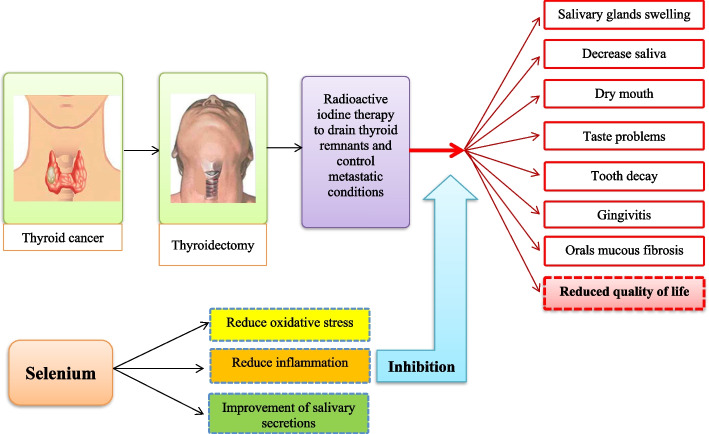
The effect of selenium on salivary glands, inflammatory factors, and oxidative stress in thyroid cancer patients treated with radioactive iodine

Selenium is an essential mineral that affects the metabolism of thyroid hormones, the antioxidant system, and the inhibition of thyroid neoplasm [29, 32, 34, 36]. Selenium can modulate the growth of thyroid cancer cells by stopping the cell cycle and inhibiting cell growth factors. Also, sufficient amounts of this mineral reduce the possibility of DNA damage [36, 49]. Selenium exerts its anti-inflammatory role by modulating inflammatory factors [50, 51]. Selenium, as one of the most important parts of antioxidant selenoproteins, is effective in improving oxidative stress factors [52–55] (Fig. 3).

Selenium exerts part of its anticancer effects by controlling redox stimuli and thus modulating the redox process [56]. A recent systematic review including 15 articles reported that selenium nanoparticles cause significant cytotoxicity against cancer cells, while normal cells are less affected [57]. Some metabolites derived from selenium, including methyl selenol, can stop the cell cycle in the G1 phase, thus being effective in inhibiting the growth of cancer cells [58].

A review study in 2020 showed that supplementation with 300 to 500 μg/day selenium for a period of 10 days to 6 months without reducing the effectiveness of radiotherapy can reduce the side effects of this therapeutic strategy in several types of cancer treated with radiation therapy. This study did not report any side effects from selenium supplementation [43].

Another review study reported that selenium supplementation reduces ageusia (loss of taste) and dysphagia and diarrhea caused by radiation therapy in various cancers. This supplement did not inhibit the therapeutic effects of radiation therapy and had no side effects [44].

Recently, in a clinical trial study, it was reported that selenium supplementation in thyroid cancer patients treated with radioactive iodine can help maintain salivary gland function. But this study only had 16 patients as a sample size. Also, the effect of selenium on oxidative stress and inflammation has not been investigated [38]. Therefore, the present study will be conducted to investigate the effectiveness of selenium supplementation in patients with PTC treated with radioactive iodine with a larger sample size (*n* = 60).

The main limitation of our protocol is that, due to ethical issues, we will not be able to stop the patients' other medications and study the effect of selenium alone. The solution we have thought of for this limitation is that we will carefully examine all the drugs received by the two groups (selenium receiving group and placebo group) at the beginning and end of the study. Then, if there is a significant difference between the two groups, we will adjust the results.

Another limitation is that due to a lack of funding, it is not possible to consider selenium blood biomarkers to assess adherence to supplements. To control this limitation, the patients will be closely monitored in such a way that the patients will be reminded daily by phone call to take the supplement during the ten days of the intervention. Also, patients return the empty can of the supplement after ten days. In addition, the potential benefits of the current research for patients are described in detail so that patients can cooperate better.

If the present study observes the effectiveness of selenium on thyroid cancer, selenium may be used as a safe supplement with a high potential in improving the symptoms of these patients in the near future.

## Trial status

Version 1, September 2021 Recruitment will be started in February 2023 and approximately will be completed in September 2023.

### Supplementary Information

**Additional file 1. ** 

## Data Availability

If the data request is in the direction of the present protocol, the corresponding author sends the data set. Otherwise, all information will remain confidential.

## Abbreviations

TC: Thyroid cancer
DTC: Differentiated thyroid carcinoma
ATC: Anaplastic thyroid carcinoma
MTC: Medullary thyroid carcinoma
CRP: C-reactive protein
NF-κB: Nuclear factor kappa light chain enhancer of activated B lymphocytes
TKI: Tyrosine kinase inhibitors
RAI: Radioactive iodine
RIT: Radioiodine therapy
TAC: Total antioxidant capacity
SOD: Superoxide dismutase
